# A Piercing Scar Over the Nose: A Rare Case of Cutaneous Pseudolymphoma

**DOI:** 10.7759/cureus.85327

**Published:** 2025-06-04

**Authors:** Shivani Wadhwa, Sanjeev Gupta, Aditi Dabhra

**Affiliations:** 1 Department of Dermatology, Fakeeh University Hospital, Dubai, ARE; 2 Dermatology, Maharishi Markandeshwar Institute of Medical Sciences and Research, Ambala, IND

**Keywords:** body piercing, complication, nose, nose piercing, pseudolymphoma

## Abstract

In this study, we explore the diagnostic challenges of distinguishing hypertrophic scars from other conditions at piercing sites, illustrated by a case report of a young woman. The case highlights the importance of considering less common conditions, such as cutaneous pseudolymphoma, which can clinically mimic hypertrophic scars. The patient presented with a persistent raised spot on her nose six months after a piercing, initially treated as a hypertrophic scar. However, a biopsy revealed cutaneous pseudolymphoma, emphasizing the need for thorough evaluation and biopsy when standard treatments fail.

## Introduction

Body piercings are a widespread cultural and cosmetic practice, with many individuals opting for them for aesthetic or personal reasons. While most piercings heal without complications, various issues can arise, ranging from minor problems to more serious pathological conditions. Infections, allergic reactions, hypertrophic scars, and keloids are common complications noted at piercing sites. These issues can typically be managed with appropriate care, such as antibiotic treatment or scar management techniques. However, less common but more serious conditions can also develop at piercing sites, including pyogenic granulomas, cutaneous sarcoidosis, lupus vulgaris, and basal cell carcinoma (BCC) [1]. Additionally, cutaneous pseudolymphoma, a benign lymphocytic infiltrate resembling lymphoma, can present at these sites [2]. These conditions may mimic normal healing responses or cosmetic imperfections, which can lead to misdiagnosis and delayed treatment. This case emphasizes the importance of not dismissing lesions at piercing sites as merely cosmetic concerns or typical scars.

## Case presentation

**Figure 1:**
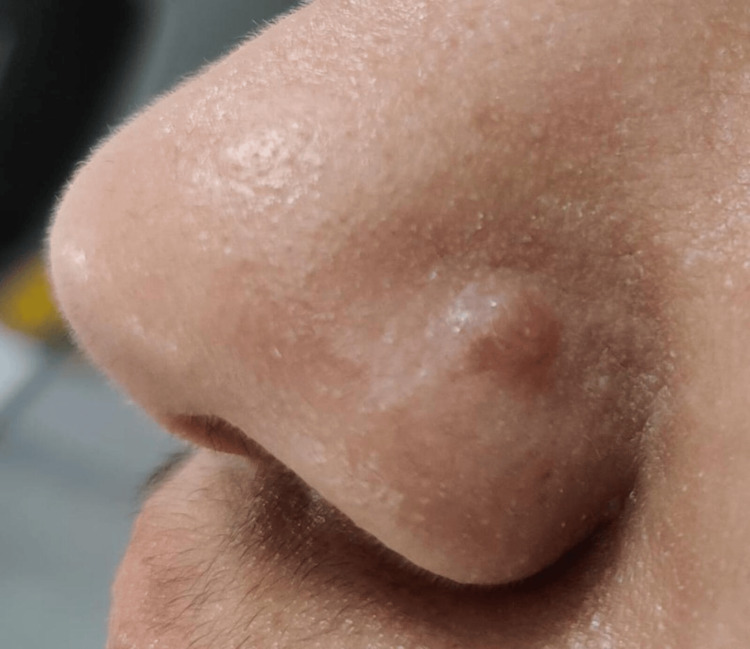
Preoperative image showing a skin-colored to slightly erythematous papule located precisely at the piercing site.

A 37-year-old Indian female presented to our dermatology outpatient department with a persistent raised spot on the side of her nose, persisting for six months. She had a history of nose piercing done with a medicated stud following usual aseptic precautions. The stud was removed by the patient owing to some discomfort in the pierced area. She noticed the lump on the pierced area in about four to six weeks after the piercing. The patient reported no associated symptoms such as itching, oozing, pain, or bleeding. She had been applying silicone and scar gel without any noticeable improvement. The patient’s medical history was unremarkable except for hypothyroidism, managed with oral levothyroxine 50 µg daily for the last few years. There was no history of any other medication intake. Clinical examination revealed a single, dome-shaped, firm, non-tender papule measuring approximately 5 mm. The papule was skin-colored to slightly erythematous and located precisely at the piercing site (Figure 1).

**Figure 2:**
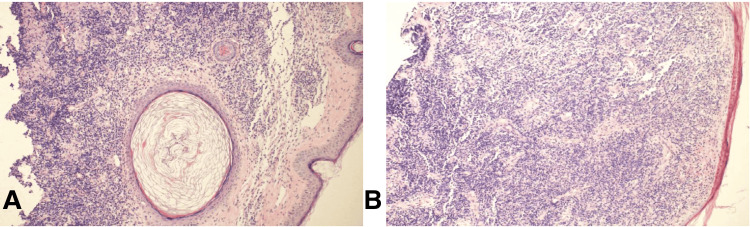
Histopathological images. A: Histopathological section showing dense lymphocytic infiltrates in the papillary and reticular dermis, with a grenz zone and folliculocentric arrangement accompanied by follicular hyperplasia (hematoxylin and eosin stain 10 x 100×). B: Diffuse infiltrate comprising small and larger lymphocytes with collections of histiocytes in the dermis (hematoxylin and eosin stain 10 × 100×).

**Figure 3:**
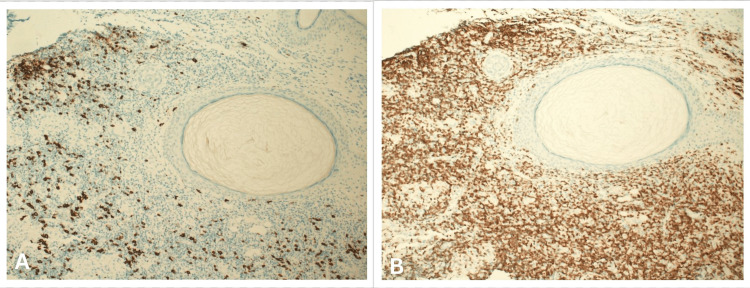
Immunohistochemistry images. A: Histopathological section showing focally CD20-positive B cells (immunostain 10 × 100×). B: Histopathological section showing a mixed lymphoid infiltrate with predominantly CD3-positive cells (immunostain 10 × 100×).

Dermoscopy findings were inconclusive. A preliminary clinical diagnosis of a hypertrophic scar was made, and the patient underwent two sessions of fractional CO₂ laser therapy. However, no significant improvement was observed, prompting reconsideration of the alternative diagnoses, such as foreign body granuloma, dermatofibroma, pyogenic granulomas, cutaneous sarcoidosis, lupus vulgaris, BCC, and cutaneous pseudolymphoma. A shave excision biopsy of the lesion was performed, and histopathological examination revealed dense lymphocytic infiltrates in the papillary and reticular dermis, with a grenz zone and folliculocentric arrangement accompanied by follicular hyperplasia. The infiltrate comprised small and larger lymphocytes with collections of histiocytes but no distinct germinal centers. No cellular atypia was observed. The epidermis was unremarkable, and no polarizable material was seen, consistent with cutaneous pseudolymphoma (Figures 2A, 2B). The immunohistochemistry studies showed a mixed lymphoid infiltrate with predominant CD3-positive and focally CD20-positive B cells, supporting the diagnosis of pseudolymphoma (Figures 3A, 3B).

Following the biopsy and diagnosis, the treated site healed without significant scarring. At the 24-month follow-up, the patient reported no relapse or recurrence of the lesion.

## Discussion

Body piercings, although widely practiced for cultural, aesthetic, or personal reasons, are not without complications. These range from hypertrophic scars, keloids, contact dermatitis, and traumatic granulomas to less common but clinically significant presentations such as pyogenic granulomas, cutaneous pseudolymphomas, and even malignancies such as BCC.

Cutaneous pseudolymphoma: an underdiagnosed entity

Pseudolymphoma refers to skin lesions that closely resemble lymphoma, primarily in histological appearance and occasionally in clinical presentation. Despite their resemblance to malignant conditions, these lesions exhibit benign biological behavior at the time of diagnosis and do not meet the diagnostic criteria for malignancy. Depending on the composition of lymphoid cells involved, pseudolymphomas can be categorized into T-cell and B-cell types. Cutaneous T-cell pseudolymphomas encompass a variety of conditions, including idiopathic cutaneous T-cell pseudolymphoma, drug-induced lymphomatoid reactions, lymphomatoid contact dermatitis, persistent nodular reactions to arthropod bites, nodular scabies, actinic reticuloid, and lymphomatoid papulosis. On the other hand, cutaneous B-cell pseudolymphomas consist of idiopathic lymphocytoma cutis, borrelial lymphocytoma cutis, tattoo-related lymphocytoma cutis, lymphocytoma at post-zoster scar sites, trauma-induced pseudolymphomas, lymphocytoma triggered by gold earrings, and certain long-standing nodular reactions to arthropod bites. The term cutaneous lymphoid hyperplasia is commonly used to refer to B-cell pseudolymphomas [3]. The condition is characterized by dense lymphocytic infiltration in the dermis, often forming germinal centers. The development of cutaneous pseudolymphoma is believed to involve an exaggerated immune response to external stimuli. Trauma or irritation from a piercing can act as a trigger, leading to localized lymphoproliferative activity. Clinically, cutaneous pseudolymphoma typically appears as solitary or multiple nodules, plaques, or papules. These lesions are often asymptomatic and slow-growing, which may delay diagnosis. Histological examination remains the gold standard for diagnosis. Features include dense, band-like lymphocytic infiltrates, often with germinal center formation. Immunohistochemical staining helps distinguish pseudolymphoma from malignant lymphomas [3].

When the causative agent of cutaneous pseudolymphoma is identified, removing it can lead to the resolution of the condition. For pseudolymphomas caused by infections, appropriate treatment of the infection is essential. In idiopathic cases, treatment may not be necessary as many lesions resolve on their own. However, surgical excision, cryosurgery, or local irradiation may be effective for some cases. Topical or intralesional corticosteroids and immunomodulators such as tacrolimus have shown promise in some instances. If lymphoma is suspected, patients should be monitored for possible extracutaneous involvement. Discontinuing any implicated medications is critical. While most lesions are asymptomatic, a short course of steroids may hasten regression, which typically occurs over one to three months. If lesions do not resolve, further investigation for a malignant condition is necessary. Other treatments that have been suggested include imiquimod, antibiotics in cases linked to *Borrelia* infection, and subcutaneous interferon-alfa injections. Photodynamic therapy and thalidomide have also been effective in certain cases. Surgical excision, including cryosurgery, may provide a cure, especially for lesions causing functional or cosmetic concerns. In cases of incomplete regression, external radiation therapy may be considered. Furthermore, treatments such as fractional resurfacing and Q-switched Nd:YAG laser have been successful in tattoo pigment-induced pseudolymphoma [4].

Hypertrophic scars versus mimics: diagnostic challenges

Hypertrophic scars are raised, erythematous lesions that remain confined within the boundaries of the original wound [5]. These scars develop due to an excessive accumulation of collagen during the wound-healing process. Unlike keloids, which extend beyond the wound margin and continue to grow over time, hypertrophic scars typically stabilize or regress naturally [6]. Their treatment options include the application of silicone gels, pressure therapy, corticosteroid injections, and laser therapies [7]. A raised lesion at a site of skin trauma, such as a piercing, often leads clinicians to suspect a hypertrophic scar initially. However, several other conditions can mimic this presentation and should be considered in the differential diagnosis.

One such condition is dermatofibroma, a benign, fibrohistiocytic proliferation typically presenting as a firm, hyperpigmented papule or nodule, often with a central dimpling on lateral compression (positive “dimple sign”). While classically seen on the lower limbs, dermatofibromas can also arise at sites of minor trauma, including piercings.

Another potential mimic is cutaneous sarcoidosis. It can present as succulent plaques or nodules that develop at trauma sites [8]. Diagnosis often relies on dermoscopic examination and histopathology, which can reveal granulomatous inflammation typical of sarcoidosis. Similarly, lupus vulgaris, the most common form of cutaneous tuberculosis, may present as slowly expanding plaques or nodules. These lesions can persist for months or years if left untreated. Diagnosis is confirmed through microbiological testing and histological examination, which reveals granulomas with caseating necrosis [9]. Although rare, BCC can also develop at piercing sites. BCC often presents as pearly papules, sometimes ulcerated or displaying telangiectasias. Given its potential for local invasion, a high index of suspicion is essential for early detection and treatment [10].

Diagnostic workup for persistent lesions at the piercing site

Diagnosing persistent lesions at piercing sites requires a comprehensive approach involving clinical, dermoscopic, and histopathological evaluations. Clinical examination begins with obtaining a detailed history of lesion onset, associated symptoms, and the patient’s response to prior treatments. Dermoscopy can be valuable in diagnosing lesions, such as pyogenic granuloma and basal cell carcinoma. Dermoscopy findings of pseudolymphoma include salmon pink to yellow orange background, follicular plugging, and a variable vascular pattern, which are similar to other granulomatous diseases such as sarcoidosis and lupus vulgaris. Histopathological examination through biopsy remains essential for an accurate diagnosis. In this case, histopathology revealed a dense lymphocytic infiltrate, effectively ruling out malignancy and confirming the diagnosis of pseudolymphoma. Regular follow-up is crucial to monitor for recurrence or development of new lesions, especially in cases of pseudolymphoma or other conditions with malignant potential.

Implications for clinical practice

This case highlights several important lessons for dermatologists and healthcare providers. A thorough evaluation, including a detailed clinical history and physical examination, is essential before initiating treatment for persistent lesions. When conventional treatments fail, performing a biopsy becomes crucial to avoid overlooking less common diagnoses such as pseudolymphoma or malignancies. While hypertrophic scars are a frequent finding, clinicians must remain vigilant for conditions that mimic their presentation, including sarcoidosis, dermatofibroma, pseudolymphoma, BCC, and lupus vulgaris. Additionally, counseling patients about the potential complications of piercings and the importance of seeking timely medical evaluation can help prevent diagnostic delays and improve outcomes.

## Conclusions

While hypertrophic scars and keloids are commonly recognized complications of piercings, it is crucial to consider less common conditions, such as cutaneous pseudolymphoma, which may mimic these presentations. This case highlights the significance of a thorough clinical evaluation and the need for biopsy when standard treatments fail to produce results. Accurate diagnosis is essential not only for effective treatment but also to alleviate the psychological and cosmetic burden on patients.

## References

[1] Abudu B, Erickson CP, Calame A, Cohen PR (2019). Basal cell carcinoma originating in a tattoo: case report and review of an uncommon complication in tattoo recipients. Dermatol Pract Concept.

[2] Laftah Z, Benton E, Bhargava K, Ross J, Millard T, Craig P, Calonje E (2014). Two cases of bilateral earlobe cutaneous pseudolymphoma. Br J Dermatol.

[3] Carswell L, Borger J (2025). Hypertrophic Scarring Keloids. https://pubmed.ncbi.nlm.nih.gov/30725743/.

[4] Trace AP, Enos CW, Mantel A, Harvey VM (2016). Keloids and hypertrophic scars: a spectrum of clinical challenges. Am J Clin Dermatol.

[5] Limandjaja GC, Niessen FB, Scheper RJ, Gibbs S (2021). Hypertrophic scars and keloids: overview of the evidence and practical guide for differentiating between these abnormal scars. Exp Dermatol.

[6] Abdelghaffar M, Hwang E, Damsky W (2024). Cutaneous sarcoidosis. Clin Chest Med.

[7] Hassan I, Ahmad M, Masood Q (2010). Lupus vulgaris: an atypical presentation. Indian J Dermatol Venereol Leprol.

[8] McDaniel B, Steele RB (2025). Basal Cell Carcinoma. https://pubmed.ncbi.nlm.nih.gov/29494046/.

[9] Bhobe M, Kakode NP, Pednekar S, Pai V (2016). Cutaneous pseudolymphoma: a case report. Muller J Med Sci Res.

[10] Bergman R (2010). Pseudolymphoma and cutaneous lymphoma: facts and controversies. Clin Dermatol.

